# Three-dimensional Virtual Reality as an Innovative Teaching and Learning Tool for Human Anatomy Courses in Medical Education: A Mixed Methods Study

**DOI:** 10.7759/cureus.7085

**Published:** 2020-02-24

**Authors:** Yasser Alharbi, Mubarak Al-Mansour, Radi Al-Saffar, Abdullah Garman, Abdulrahman Alraddadi

**Affiliations:** 1 Basic Medical Science, College of Medicine, King Saud Bin Abdulaziz University for Health Sciences, Jeddah, SAU; 2 Adult Medical Oncology, Princess Noorah Oncology Center / College of Medicine, King Abdulaziz Medical City, Ministry of National Guard Health Affairs-Western Region / King Saud Bin Abdulaziz University for Health Sciences, Jeddah, SAU; 3 Anatomy, College of Medicine, Imam Abdulrahman Ibn Faisal University, Dammam, SAU; 4 Basic Medical Science, College of Medicine, King Saud Bin Abdulaziz Univesity for Health Sciences, Riyadh, SAU

**Keywords:** human anatomy, medical education, 3d-vr, virtual reality, medical curriculum

## Abstract

Introduction

Poor knowledge retention is one reason for medical student attrition in learning and has been a huge concern in medical education. Three-dimensional virtual reality (3D-VR)-based teaching and learning in medical education has been promoted to improve student learning outcomes. This study aimed to determine the effectiveness of 3D-VR in knowledge retention in human anatomy courses as compared to traditional teaching methods among medical students.

Methods

A convergent mixed methods design was utilized to evaluate learning outcomes in terms of short- and long-term knowledge retention scores among students using 3D-VR and those using traditional models and to describe students’ experiences and views of the use of 3D-VR as a teaching and learning tool.

Results

Male students who used the 3D-VR tool had significantly higher short- and long-term knowledge scores than males who used the traditional methods. Meanwhile, females who used traditional methods showed significantly higher short-term knowledge scores than females who used 3D-VR.

Conclusion

Medical students described 3D-VR as a learning tool with a great deal to offer for learning human anatomy as compared to traditional methods. Therefore, we recommend adding the use of 3D-VR in the anatomy curriculum. However, several 3D-VR limitations were also identified, which may hinder its utilization for teaching and learning. These concerns must be addressed before 3D-VR tools are considered for implementation in medical education human anatomy courses.

## Introduction

There is apparent attrition in medical students’ knowledge of human anatomy, which needs to be evaluated and reviewed. Poor knowledge retention in human anatomy courses is thought to be one of the reasons for this problem, which is a huge concern in medical education [1-5].

The development of reformed curricula in medical schools and allied health institutions has provided an opportunity for improved student knowledge acquisition in human anatomy courses through the incorporation of technology into teaching in the form of computer-generated simulations [6]. Thus, the three-dimensional virtual reality (3D-VR)-based teaching and learning in medicine has been promoted to improve student learning outcomes.

The design of 3D software models has paved the way for the use of multiple orientations of anatomical structures and the simplification of complex tasks in the teaching and learning process as compared to traditional teaching methods [7-10]. These changes have reduced the number of teaching hours and lab sessions devoted to human anatomy. This also allows students more individual study and better mastery of human anatomy [3].

Students should be acquainted with the spatial relationships around anatomical structures rather than just their appearances and functions. This is a challenge in traditional classes in which students use two-dimensional (2D) pictures from anatomical textbooks and articles as references [4]. In contrast, 3D-VR offers students the potential to build a strong foundation in human anatomy because it facilitates spatial visualization to maximize the input and internalization of the anatomical structures.

The difficulties that medical students experience in classroom and practical lessons while using 2D diagrams from reference materials to visualize the 3D dynamism of anatomical features can affect their knowledge, retention, and understanding of certain functions of these structures [11-14]. For example, a student might not recognize the caudate lobe of the liver if the structure is moved to a different position. As such, 3D-VR provides students the ability to manipulate structures in various ways to better understand anatomical concepts. Thus, in learning anatomical structures, the visual-spatial ability of the learner can be enhanced by the mental manipulation of the desired outcomes in three dimensions [12].

The aspect of visualization and mental influence as the goals of 3D structures while correctly identifying anatomical structures is a vital skill in medicine [13]. This enables students to appreciate the relationships between spatial features and mentally manipulate structures in 3D, thus improving their knowledge retention by developing their visual-spatial ability, individual control, and search skills [8].

Despite the benefits of 3D tools for student learning and knowledge acquisition, some studies have found no significant differences between modern 3D learning resources, traditional 2D approaches, and dissections in terms of student human anatomy knowledge acquisition [4,5,7-14]. However, studies conducted in different universities in Saudi Arabia suggested 3D-VR as an excellent teaching methodology to improve student human anatomy knowledge retention [15-20].

This study aimed to determine the effectiveness of 3D-VR in knowledge retention in human anatomy courses as compared to traditional teaching methods among medical students.

## Materials and methods

Study design and setting

This study used a convergent mixed-methods design conducted in the human anatomy laboratory of the College of Medicine of King Saud Bin Abdulaziz University for Health Sciences in Jeddah, Saudi Arabia.

Plastinated human anatomy models and 3D-VR human anatomy models installed on iPads were utilized as study interventions for the control group and the experimental group, respectively. The effectiveness of these teaching tools on students’ short- and long-term knowledge retention was assessed. Subsequently, focus group discussions were conducted to understand students’ experiences and views about the use of 3D-VR as a teaching and learning tool for human anatomy courses.

Sample size

The study population consisted of 182 third-year medical students of the foundation block (i.e., 3rd Year, Phase II). The sample sizes for two unequally sized groups (i.e., male and female) were calculated using Openepi, an open-source software for epidemiologic statistics, which was accessed from http://openepi.com/SampleSize/SSMean.html. For the 108 male medical students, a calculated difference of 2.5 was obtained for a sample size of 104. Then, they were divided into the control group (n=52) and the experimental group (n=52). For the 74 female students, a calculated difference of 3.5 was obtained for a sample size of 66. Then, they were also divided into the control group (n=33) and the experimental group (n=33). The standard deviation (SD) between the two groups was 9. Alpha was taken at 5% with the power of the study at 0.80.

Interventions

Plastinated models of bones, joints, and ligaments were used as the teaching tools for the control group (a total of 10 different human anatomy models), while human anatomy modules on a 3D-VR software platform installed on iPads were utilized for the experimental group. Prior to the interventions, two-hour lectures were conducted for the control and experimental groups by an expert anatomist.

Knowledge retention assessment

Pre- and post-tests were administered to both groups to assess the students’ short-term knowledge retention. After four weeks, a follow-up test was given to assess the students’ long-term knowledge retention. All tests were administered through the objective structured practical examination (OSPE).

OSPE implementation

OSPE consists of 10 slides. Each slide has two questions for a total of 20 questions. The total OSPE time was 20 minutes. All questions were validated by two expert anatomists for construct and content validity.

Focus group discussions

The focus group discussions, consisting of 72 medical students divided into 13 groups, were facilitated by an expert anatomist. These students were voluntary participants from the experimental group. The group discussions about the students’ personal experiences and the views of the use of 3D-VR as a teaching and learning tool were audiotaped and transcribed verbatim.

Data management and analysis

Quantitative data were encoded and analyzed using Statistical Packages for Social Sciences (SPSS) version 23.0 (IBM Corp., Armonk, NY). Descriptive statistics, such as mean and SD, were used for the pre-, post-, and follow-up test scores. A paired t-test was used to determine whether there was a significant difference between the pre- and post-test scores to determine the students’ short-term knowledge retention. A paired T-test was also used to determine whether there was a significant difference between the post- and follow-up test scores to determine students’ long-term retention of knowledge. Moreover, an independent t-test was used to compare the control and experimental groups on all scores. A p-value of < 0.05 was considered statistically significant.

Thematic analysis was performed on the qualitative data gathered from the focus group discussions on the students’ personal experiences and views about the use of 3D-VR as a teaching and learning tool. Themes and subthemes were identified and the meanings of the content were described.

## Results

Quantitative findings

There were 104 male and 66 female third-year medical students included in the final analysis (n=170). In three tests, the knowledge scores of the 3D-VR male and female groups were relatively higher than those of the traditional method groups (Tables 1-2). However, after further analysis, the post-test scores (i.e., short-term knowledge retention scores) of the 3D-VR male group were significantly higher than males of the traditional method group (traditional = 5.6±1.61 vs. 3D-VR 7.3±7.3; p < 0.001). The 3D-VR male group also had significantly higher follow-up scores (i.e., long-term knowledge retention scores) than males of the traditional method group (traditional= 6.3±2.05 vs. 3D-VR= 7.7±1.80; p < 0.001) (Table 1). Although the knowledge scores in three tests of the female 3D-VR and female traditional method groups were higher than those of the males (Tables 3-4), only the post-test scores (i.e., short-term knowledge retention scores) of the females in the traditional method group were significantly higher than those of the males (female = 7.6 vs. male = 5.6; p < 0.001) (Table 3).

**Table 1:** Knowledge score between the traditional and 3D-VR male groups Three-dimensional virtual reality (3D-VR)

Tests	Groups	N	Mean Score	SD	p-value
Pre-test	Traditional	52	5.9	1.37	0.078
	3D-VR	52	6.5	2.05	
Post-test	Traditional	52	5.6	1.61	<0.001
	3D-VR	52	7.3	1.80	
Follow-up test	Traditional	52	6.3	2.05	<0.001
	3D-VR	52	7.7	1.80	

**Table 2:** Knowledge score between the traditional and 3D-VR female groups Three-dimensional virtual reality (3D-VR)

Tests	Groups	N	Mean Score	SD	p-value
Pre-test	Traditional	33	5.9	1.77	0.070
	3D-VR	33	6.5	1.84	
Post-test	Traditional	33	5.6	2.39	0.02
	3D-VR	33	7.3	2.21	
Follow-up test	Traditional	33	6.3	1.83	0.01
	3D-VR	33	7.7	2.05	

**Table 3:** Knowledge score between males and females using the traditional method Three-dimensional virtual reality (3D-VR)

Tests	Gender	N	Mean Score	SD	p-value
Pre-test	Male	52	5.9	1.37	0.037
	Female	33	6.6	1.77	
Post-test	Male	52	5.6	1.61	<0.001
	Female	33	7.6	2.39	
Follow-up test	Male	52	6.3	2.05	0.019
	Female	33	7.3	1.83	

**Table 4:** Knowledge score between males and females using the 3D-VR method Three-dimensional virtual reality (3D-VR)

Tests	Gender	N	Mean Score	SD	p-value
Pre-test	Male	52	6.5	2.05	0.99
	Female	33	6.5	1.84	
Post-test	Male	52	7.3	1.80	0.48
	Female	33	7.6	2.21	
Follow-up test	Male	52	7.7	1.80	0.62
	Female	33	7.5	2.05	

Qualitative findings

Six themes emerged from the focus group discussions about the use of 3D-VR as a tool for the teaching and learning of medical students in human anatomy courses: 1) 3D-VR features aid the learning process (display and presentation, usage and purpose, complete source of information); 2) 3D-VR information is very available and accessible; 3) 3D-VR provides a real-like learning experience that facilitates teacher-student learning engagement; 4) 3D-VR can be used as a complementary learning tool with traditional cadaver models; 5) 3D-VR has usage limitations (difficult to use and device functionality limitations); and 6) 3D-VR use poses health risks (eye strain and headache).

Theme I: 3D-VR Features Aid the Learning Process

Medical students described how using 3D-VR allowed for the better visualization of anatomical parts and their surrounding structures, leading to better understanding. This theme had three categories. The first category was “display and presentation.” Students described how the 3D-VR application was designed so that students could completely view anatomical parts and structures in whatever angle they preferred. According to one student, “the 3D-VR offers a panoramic view of the organ and its surrounding structures from all angles” (Group 1: Male medical student 2). Another student stated the tool allowed her “to have a better focus on the subject organ by viewing it in different anatomical planes like anteriorly, posteriorly” (Group 3: Female medical student 3).

The second category was “usage and purpose.” Medical students thought using 3D-VR helped them clearly identify and differentiate anatomical structures from the surrounding structures, improving their comprehension and memory retention about human anatomy. The 3D-VR application provides the complete presentation of an organ and its surrounding structures for a better understanding of their associated functionality and differences. According to one student, “it allows me to see the details of muscle and bones ... It also allows me to manipulate the organ by separating it from other structures or surrounding organs” (Group 3: Female medical student 3). Another student stated, “the 3D-VR brings everything together, such as the origin of the muscle, its insertion, and functions” (Group 6: Male medical student 3). Meanwhile, a third student found that the 3D-VR enabled him “to see every organ specifically, like the muscle and the nerves. It also has a visual illusion to see anatomical images as real-like human organs … that is, to differentiate between nerves and arteries easily” (Group 9: Male medical student 6).

The third category was “having the complete source of information.” Students described the 3D-VR as a learning tool packed with all the information needed for their studies, helping them improve their comprehension of the topic. One student stated that “it provides us the full detail of the organ and its surrounding organs” (Group 9: Male medical student 5). Meanwhile, another student described how “every structure has a link information. For example, if you want an information of an organ like its blood supply and nerves, the organ is provided with all necessary information and you don’t need to read books” (Group 2: Male medical student 5). Another student provided additional commentary: “It is more convenient for me to use an iPad and specify any anatomical parts and structures I want. For instance, if I have the whole human anatomy, then I can hide some parts and pinpoint a specific part. Thus, this will help to enhance my comprehension about the anatomical part or structure being studied." (Group 7: Male medical student 5).

Theme II: The Availability and Accessibility of Information

Medical students preferred using a learning tool that provides information that is completely accessible and available. Since 3D-VR is a software program that can be installed on smartphones and tablets, it provided a great deal of convenience to the students because they could search for information anytime and anywhere. One student remarked, “there is no time and usage limitation. Once I have the program in my iPad and it’s fully charged, I can have a review at any given moment” (Group 1: Female medical student 1). Another said, “I can take my time and see the structure with nobody saying, ‘speed-up or hurry-up’” (Group 4: Male medical student 1). Regarding accessibility, others added that “students will have the equal access from everywhere” (Group 10: Male medical student 4) and “we can learn from anywhere, not just in the lab” (Group 1: Male medical student 3).

Theme III: Real-like Learning Experience that Facilitates Teacher-Student Learning Engagement

Since 3D-VR provides a complete functional representation of an organ through a structural image that mimics the real human organ, it helped the students have real-life, self-directed learning experiences. One student said, “it mimics the reality as much as possible. Ah, I can see the nerves, I can see the muscle, I can remove and add the nerves that are superficial and deep. It’s very nice” (Group 7: Male medical student 1). Another added, “it shows certain movements of joints and how the muscle is exactly moving this part. Moreover, this will allow you to explore more of its functionality” (Group 4: Male medical student 5). Students also felt 3D-VR facilitated teacher-student learning engagement through active learning “because the teacher and students both shares information” (Group 7: Male medical student 4) and because 3D-VR “promotes better student engagement and focus on the concept” (Group 10: Male medical student 5).

Theme IV: Complementary Use of 3D-VR and Traditional Cadaver Methods for Comprehension and Memory Retention

Medical students suggested that traditional methods, such as using cadavers, would complement the use of the 3D-VR method for a better teaching and learning process. Specifically, they felt the hands-on experience of working with cadavers combined with the use of 3D-VR would enhance their comprehension and memory. According to one student, “I think if we are just using 3D-VR and without the cadaver, I think it’s not good for teaching” (Group 10: Male medical student 5). Another added, “I can visualize and touch the cadaver by my hand. In my opinion, it will enhance my memorization as compared to 3D-VR alone” (Group 2: Female medical student 2). A third student said, “you have to compliment the 3D-VR with the cadaver at the end of the day to know everything” (Group 1: Male medical student 4).

Theme V: 3D-VR Usage Limitations

The use of 3D-VR as a teaching and learning tool was found to have several limitations, such as difficulties using the application and battery power shortages during use. These issues affected students’ focus and ability to catch up on lectures. One student relayed, “I had a difficulty in using the apps in catching up with the lecture” (Group 3: Male medical student 5), while another noted, “the usage of application software is dependent on the battery of the device” (Group 4: Male medical student 5). However, the students recognized that practice using the application was necessary to appreciate its full capabilities and benefits. One student commented that “we need to practice using this app” (Group 4: Male medical student 3).

Theme VI: Health Risks

Some medical students experienced eye strain, eye pain, and headaches while using the 3D-VR application, which affected their concentration. These issues could be related to direct eye exposure to high-intensity screen lights. One student said, “that is harmful for your eyes, and it will affect your study concentration” (Group 5: Male medical student 3) while another added that “staying for long hours with my eyes on the iPad screen can cause a headache for me” (Group 9: Male medical student 1).

## Discussion

In recent years, various teaching tools, such as computer-aided learning, web-based technologies, 3D-VR models, and mobile devices applications, have been developed to improve the efficiency of teaching human anatomy [21-23]. These tools have accomplished several tremendous outcomes for teaching human anatomy [3-4,12,15-16]. Of the teaching tool innovations, 3D-VR is the newest, and the exact utility of this technology in student knowledge retention has not been fully evaluated. Thus, this study aimed to determine the effectiveness of 3D-VR in student knowledge retention as compared to traditional methods.

Recently, 3D-VR has been used in medical education for illustrating and demonstrating human body structures within a virtual environment [8,10,13]. This enhances knowledge retention because it involves multisensorial learning that promotes student engagement in contrast to traditional methods such as the use of cadavers or plastinated models [5]. The qualitative findings of the present study show that using 3D-VR facilitates teacher-student learning engagement. Several students expressed positive experiences using 3D-VR in learning human anatomy; however, others thought using the traditional cadaver model as a complement to 3D-VR would lead to a better teaching and learning process. The effectiveness of 3D-VR as a teaching and learning tool was observed among the male medical students. Their use of 3D-VR led to relatively higher short- and long-term knowledge scores as compared to those who used the traditional plastinated models. However, females who used the traditional plastinated anatomical models had higher short-term knowledge scores than the males who used 3D-VR and the traditional plastinated anatomical models.

As this study also revealed that 3D-VR had no significant association with short- or long-term knowledge retention among female medical students, further study is recommended in this area. In addition, the interval time of four weeks between the intervention and the long-term evaluation is suggested for revalidation and comparison with longer interval durations to test its reliability. Evaluation of the knowledge retention of the human anatomy needs a certain time interval between the intervention and the evaluation period. It has been suggested that to get reliable results, it requires a time interval of six months [23]. In the present study, a four-week interval was used instead of six months because of the following considerations: first, the duration of the study was only two months due to the heavy academic schedule of the medical students. Second, the foundation block has an eight-week duration; therefore, the middle of the block was the best option to conduct the assessment and evaluation.

Although 3D-VR has been shown to be effective for teaching and learning human anatomy, in particular, and for knowledge retention among the study participants and in various medical colleges in Saudi Arabia [1], the present study identified several limitations of 3D-VR such as the students’ lack of familiarity with how to use the application, the application’s effect on battery operations, the lack of hands-on experience, and the preference of some students to continue to use a cadaver or plastinated model as a complement to 3D-VR for better comprehension and knowledge retention. These concerns must be addressed before 3D-VR teaching and learning tools are considered for implementation in medical education human anatomy courses.

This study provides scientific evidence of medical students’ acceptance of the use of 3D-VR as a teaching and learning tool for human anatomy courses and its effectiveness in improving student learning and knowledge retention.

## Conclusions

In this study, 3D-VR was shown to be an effective learning tool for short- and long-term knowledge retention among male medical students while the traditional method was shown to be effective for short-term knowledge retention among female medical students. Medical students described 3D-VR as a learning tool, with a great deal to offer in learning human anatomy as compared to traditional methods. This tool helped improve their comprehension and knowledge retention, promoted teacher-student learning engagement, and helped students have real-like, self-directed learning experiences. However, traditional methods, such as the use of cadavers, were suggested as complementary tools to 3D-VR for teaching and learning. Since the use of 3D-VR in the teaching and learning of human anatomy is new, several limitations for its use were found, which may hinder its utilization for teaching and learning. Therefore, teachers and students should attempt to fully learn how to use this tool to overcome some of these limitations and understand the true benefits of this innovative learning tool.
